# Defining Abnormal Fetal Growth and Perinatal Risk: Population or Customized Standards?

**DOI:** 10.1371/journal.pmed.1002229

**Published:** 2017-01-31

**Authors:** Sarah J. Stock, Jenny Myers

**Affiliations:** 1 Tommy’s Centre for Maternal and Fetal Health, MRC Centre for Reproductive Health, University of Edinburgh Queen’s Medical Research Institute, Edinburgh, United Kingdom; 2 School of Women’s and Infant’s Health, University of Western Australia, Crawley, Western Australia, Australia; 3 Maternal & Fetal Health Research Centre, University of Manchester, Manchester Academic Health Science Centre, Manchester, United Kingdom

## Abstract

In January's Guest Editorial, Sarah Stock and Jenny Myers discuss approaches to fetal and neonatal growth assessment.

Appropriate fetal growth and development in utero is essential for newborn health and lifelong well-being. Both fetal growth restriction (in which the fetus does not achieve its growth potential, usually because of placental insufficiency) and macrosomia (excessive in utero growth, frequently associated with maternal obesity and/or diabetes), are associated with stillbirth, neonatal morbidity and mortality, and long-term risks to health [1,2]. An aim of obstetric care is to detect fetuses at risk of complications from fetal growth disorders and intervene to reduce the risk. In reality, the only current effective intervention to prevent stillbirth is delivery of the fetus at a point when the risks of continuing the pregnancy are thought to outweigh the risks of birth [3]. It is presumed, although not proven, that timely delivery also minimizes neonatal and longer-term morbidities and mortality.

There is substantial variation in the fetal and newborn growth charts used to detect growth deviations. Although there is broad recognition that appropriate unified standards are needed, two different approaches to fetal and neonatal growth assessment have been advocated. One is based on the premise that fetal growth is strongly influenced by genetic factors and adjusts for this by creation of growth charts customized for specific phenotypic traits such as maternal ethnicity, height, and weight [4]. The other is to define optimal fetal growth standards at a population level. This is based on the theory that growth potential is similar across populations, and deviation from this norm indicates deprivation or other environmental influences rather than inherent biological differences [5]. Although there are enthusiasts for each approach, they have yet to be directly compared in a clinical trial, and it is not clear if either is superior for detecting the fetus or infant at risk of morbidity because of growth disorders. We are thus reliant on evidence from observational studies. Two articles published in *PLOS Medicine* contribute to the evidence base regarding the use of population charts for detection of fetal growth disorders and how best to determine risk of complications [6,7].

Kiserud et al. [6] from the World Health Organization (WHO) performed a prospective cohort study in around 1,400 women in ten middle- and high-income countries with the aim of creating population standard fetal growth charts. The work followed on from the production of WHO international growth standards for infants and children up to age five years, based on the concept that growth potential is similar across all populations. However, although the authors produced fetal growth charts for international use, they did find significant variation in growth between countries, with a difference in median birth weight of approximately 300 g in countries with comparable maternal complication rates; this is significantly greater than the difference between male and female infants. Furthermore, growth was influenced to a small extent (1%–2%), by maternal age, height, weight, and parity, as well as by fetal sex; thus, the authors conclude that the charts may need to be adjusted for local use.

While the aim of Kiserud et al. was to produce charts defining optimal growth, the protocol was amended at the analysis stage, and women with conditions known to influence fetal growth such as pre-eclampsia (which is associated with fetal growth restriction) and gestational diabetes (which causes fetal overgrowth) were included. The numbers of such women were small, and in a sensitivity analysis the effects of their inclusion were minimal, but nevertheless these fetuses seem unlikely to represent “optimal growth.” The lowest birth weight babies in the cohort were also included in the analysis, despite recognition that some of these babies would be pathologically growth restricted rather than physiologically small. Kiserud et al. concluded that by including complicated pregnancies, their charts would better mirror the populations for which they are intended. The alternative approach could also be problematic—if only “normally grown” babies were included, and babies below a certain threshold were excluded, percentiles would be shifted upwards. Different approaches to define normality may be one explanation for the divergent findings of Kiserud et al. compared to another recent study that aimed to create population standards from serial measurements of women with normal pregnancies [5]. In the INTERGROWTH-21^st^ project, Papageorghiou et al. excluded women with obstetric conditions that may influence fetal growth and saw less variability in fetal growth between the eight included countries [5, 8]. In contrast, a recent United States study identified significant differences in estimated fetal weight between different racial groups living in the US [9].

Whichever growth charts are utilized, arbitrarily defined cutoffs of fetal growth below the 10th and above the 90th centiles are commonly used to define the “fetus at risk” and trigger surveillance of fetal well-being and/or delivery. Iliodromiti et al. [7] challenge this dogma by using Scottish population data to determine the thresholds of birth weight that are associated with an increased risk of stillbirth and infant death (death up to one year of age) and neonatal morbidity (admission to neonatal unit and low Apgar score). They suggest that use of 10th and 90th centile cutoffs are too restrictive and that in singleton pregnancies at term (37 weeks to 43 weeks gestation), birth weight at or below the 25th centile or birth weight at or above the 85th centile are more appropriate indicators of increased risk. An important consideration for the application of these centile thresholds, however, is that they were derived from birth weight population data rather than intrauterine fetal growth data. Birth weight centiles are inevitably shifted downward when compared to fetal growth centiles, as a larger proportion of babies born at early term gestations (37–38 weeks) will be growth restricted than those remaining in utero. This is demonstrated by the difference in birth weight centiles and fetal growth centiles presented in the WHO study [6], where there is a 350 g difference in the 5th centile at 37 weeks between the birth weight centile and the population centile charts. Thus, the weight of an infant born at 37 weeks, for example, might be assigned a higher centile on a birth weight chart than on a fetal growth chart. This may explain why an elevation in risk was identified at the 25th centile in the Scottish population data.

It is also of note that in the Scottish population dataset used, the gestation recorded for stillbirths reflects the gestation at delivery, not the gestation at which the baby died [10]. A delay of a few days between diagnosis and delivery of stillbirths is common. As birth weight centiles were adjusted by gestation (in completed weeks), there is potential for systematic underestimation of the birth weight centiles of the stillbirths if they actually died in the preceding week. This may have shifted the birth weight centile associated with an increased risk of stillbirth to the left (downward). However, despite the limitations of the data used for this study, the findings clearly suggest that the wrong birth weight targets are being used to identify the fetus at risk. It seems likely that a greater proportion of at-risk babies with an abnormal growth trajectory but birth weight within the previously defined normal range will be captured by the broader criteria of Iliodromiti et al. The absolute risks of complications are low, however, presumably because the majority of babies included are actually normally grown, albeit smaller or larger than average.

Iliodromiti et al. then attempted to see if use of the GROW birth weight package [11], which customizes birth weight for parity, ethnicity, height and weight, might improve detection of babies at risk. No improvement was seen with partial customization for maternal parity and height. Unfortunately, analyses were limited by the available data, so full customization with inclusion of weight was not possible. Furthermore, there was imputation of nearly 20% of missing variables for height, and all ethnicity was presumed to be white (based on national statistics showing 98% of the population to be White British), although analysis in an independent United Kingdom pregnancy cohort found this would be unlikely to have significantly changed findings. This means that the potential value of full customization cannot be refuted by this study.

Iliodromiti et al. expand on the implications of their findings by calculating that by ultrasonographic estimation of fetal weight in utero and offering delivery to women outside of the 25th to 85th centile (rather than the traditionally used 10th and 90th centiles), an additional 1,143 deliveries would be required to prevent one fatal event (422 additional deliveries at or below 25th centile; and 721 additional deliveries at or above the 85th centile). These estimates, like any estimates of the potential benefits of growth charts, are based on two assumptions. Firstly, there is an assumption that ultrasound-based estimation of fetal weight can accurately detect low- and high-birth-weight babies. This is optimistic. Although estimated fetal weight and birth weight are correlated, in a prospective study, ultrasound only had a sensitivity of 57% for detection of babies born below the 10th centile, even when a policy of universal scanning was implemented in ideal clinical study settings [12]. Secondly, there is an assumption that delivery will reduce fatalities. It is logical to assume that delivery will prevent antepartum stillbirths. However, the hypothesis that delivery will reduce intrapartum stillbirths and infant deaths in high- and low-birth-weight infants is untested, and absence of harm from an increase in early-term delivery needs confirmation. Although Iliodromiti et al.’s suggestion that offering delivery to selected women based on growth parameters should reduce unnecessary intervention seems logical, other papers have estimated a similar number of deliveries to prevent one stillbirth with universal elective delivery at term (in the region of 1,000) [3, 13]. What level of intervention (and potential associated risk) would be acceptable to women and caregivers to prevent one baby’s death is unknown.

That fetal growth has a genetic component is not in question—the fact that fetal sex is a determinant of growth potential illustrates this. However, the amount by which obstetricians can (and should) adjust for genetic variation across populations when assessing fetal growth is unclear. Both of the papers in this issue support the concept that robustly developed population growth standards are appropriate for the diagnosis of fetal growth disorders but that thresholds of risk that are relevant to local populations should be considered. Whatever method is used, the benefits of detecting fetal growth disorders can only be realized if we can effectively reduce risk of complications. At the moment, delivery is the only way of doing this. Even using optimized charts and thresholds, we are likely to overintervene in many normal cases to prevent complications in the few. Ultrasound assessment of fetal growth has limitations, and better methods of risk prediction are needed to prevent death and disability in babies.
